# Beyond fluid therapy to prevent post-endoscopic retrograde cholangiopancreatography: is there a place for albumin?

**DOI:** 10.1093/bjsopen/zrae159

**Published:** 2025-01-21

**Authors:** Lucía Guilabert, Enrique de-Madaria

**Affiliations:** Department of Gastroenterology, Dr Balmis General University Hospital-ISABIAL, Alicante, Spain; Department of Gastroenterology, Dr Balmis General University Hospital-ISABIAL, Alicante, Spain

The main complication of endoscopic retrograde cholangiopancreatography (ERCP) is post-ERCP acute pancreatitis (PEP). Different interventions have been studied to prevent the occurrence of PEP, such as rectal non-steroidal anti-inflammatory drugs (NSAIDs), pancreatic duct stenting, and the use of aggressive fluid resuscitation based on lactated Ringer’s solution (LR)^1^. However, ductal stenting is not always feasible and rectal NSAIDs are not universally available. For this reason, the most common approach is aggressive fluid therapy with LR. Nevertheless, it should be noted that aggressive fluid therapy has been associated with fluid overload in acute pancreatitis management^2^ and may have a detrimental effect on patients.

In this issue of *BJS Open*, Shatsnimitkul *et al*.^3^ hypothesized that, by replacing part of the volume of LR with albumin, the risk of fluid overload could be reduced, while maintaining its prophylactic effect against PEP. For this purpose, a randomized clinical trial was conducted, assigning ERCP patients to either: a bolus of LR (15 ml/kg) with 600 ml of LR replaced by 50 ml of 20% human albumin 1 h pre-procedure, followed by an aggressive infusion rate (3 ml/kg/h) during ERCP and for 8 h post-procedure; or a moderate infusion rate of LR (1.5 ml/kg/h peri-procedure).

However, Shatsnimitkul *et al*.^3^ could not demonstrate a reduction in the rate of PEP with the aggressive LR + albumin combination compared with moderate LR alone (6.7% *versus* 6.3% respectively) or a benefit in fluid overload reduction, even in the subgroup of high-risk patients.

These findings challenge recently published results and recommendations advocating for aggressive fluid therapy with LR to reduce the incidence of PEP. Unlike a study with positive results that focused on patients at high risk of developing PEP^4^, the Shatsnimitkul *et al*.^3^ trial included ‘all-comers’, who are likely to have a lower rate of PEP. This may explain the negative results, suggesting aggressive hydration may help prevent PEP in high-risk populations but not universally in ERCP patients. Similarly, the FLUYT trial^5^ demonstrated no added benefit of aggressive fluid therapy in patients already receiving prophylactic NSAIDs, indicating its protective effect may not apply to all ERCP scenarios.

Also, Shatsnimitkul *et al*.^3^ found a higher rate of PEP in the aggressive LR + albumin arm than assumed when calculating the sample size based on the results of a previous study (6.7% *versus* 3.0%^4^), which could also affect the possibility of proving a difference.

Another aspect to consider is that, in the study by Park *et al*.^4^, no difference in the rate of PEP was observed when comparing aggressive normal saline with standard LR. This underscores that the prophylactic effect against PEP is probably not solely due to the volume administered but to the type of fluid used, specifically LR. LR has previously been shown to have anti-inflammatory effects in acute pancreatitis^6^. In the Shatsnimitkul *et al*.^3^ trial, substituting part of the LR with albumin may have diminished its preventive effect.

The protective role of LR was also highlighted in the study by Patel *et al*.^7^, with a lower rate of PEP (4% *versus* 11% respectively) when comparing aggressive fluid therapy with LR with aggressive fluid therapy with normal saline in patients at high risk of developing PEP. However, the difference was not statistically significant, likely due to not reaching the estimated required sample size.

Based on the findings of the Shatsnimitkul *et al*.^3^ trial, it could be concluded that there is no place for albumin in preventing PEP. Additionally, it does not reduce the risk of fluid overload, which, in itself, does not appear to be significant, as fluid therapy in PEP prevention is administered over a short interval, as demonstrated in different trials^4,5,7^.

Further studies are required to establish robust evidence on the actual role of high-volume fluid therapy, different types of fluids, and other potential preventive strategies for PEP, considering subgroups of populations with varying risk levels and the role of summative strategies.
